# Validity and reliability of the Brazilian Portuguese version of the Florida Patient Acceptance Survey for patients with implantable cardioverter defibrillators

**DOI:** 10.1016/j.mex.2023.102272

**Published:** 2023-06-27

**Authors:** Katia Regina Silva, Roberto Costa, Flávio Rebustini, Giovanna Regina Garcia de Oliveira Melo, Laísa de Arruda Silva, Sarah Caroline Martins Saucedo, Samuel Sears

**Affiliations:** a Unidade de Estimulacao Eletrica e Marcapasso, Instituto do Coracao (InCor) do Hospital das Clinicas da Faculdade de Medicina da Universidade de Sao Paulo, Sao Paulo, SP, Brasil; b Department of Gerontology (EACH), University of Sao Paulo, Sao Paulo, Brazil; c Department of Psychology and Cardiovascular Sciences, East Carolina University, Greenville, NC, United States

**Keywords:** Implantable defibrillator, Arrhythmias, Patient acceptance, Quality of life, Psychometry, Cultural adaptation, Patient-reported outcomes measures, *Psychometrics properties of the Florida Patient Acceptance Survey*

## Abstract

Device acceptance is a crucial factor in identifying implantable cardioverter defibrillator (ICD) patients at risk for psychosocial distress and unfavorable quality of life outcomes. The purpose of this study was to examine the evidence of the validity of internal structure (construct) and reliability of the Florida Patient Acceptance Survey (FPAS) in a sample of ICD patients, comparing the psychometric indicators of the complete (FPAS-18 item) and abbreviated (FPAS-12 item) versions. The sample included 151 participants (97 males, mean age of 55.7 ± 14.1 years) who completed the cross-culturally adapted version of the FPAS instrument for the Brazilian context. The psychometric properties of both versions of the FPAS instrument were evaluated by two distinct approaches:

• Exploratory and confirmatory factor analysis: used to test the internal structure of the instrument
• Cronbach's Alpha and McDonald's Omega: used to determine the reliability of the instrument

The two versions of the FPAS-Br instrument showed consistent evidence of internal structure validity and reliability. However, the FPAS-Br 12-item showed a better psychometric adjustment, confirmed by the analysis of the quality indicators of the models.

## Graphical abstract

**Figure:**
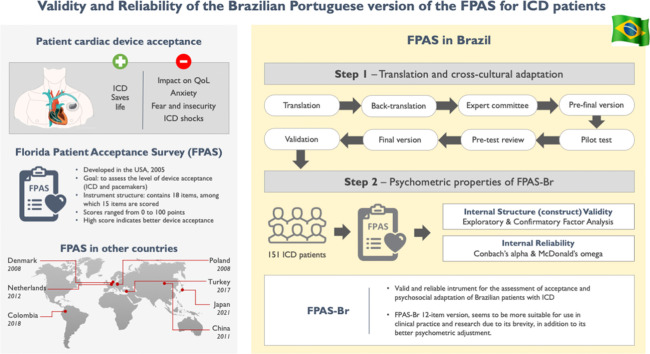

Specifications table

**Table:** 

**Subject area**	Medicine and Dentistry
**More specific subject area**	*Psychometrics*
**Name of your method**	*Psychometrics properties of the Florida Patient Acceptance Survey*
**Name and reference of original method**	*NA*
**Resource availability**	*NA*

## Method details

### Context and significance

Among cardiac implantable electronic devices, implantable cardioverter defibrillators (ICDs) are the ones most likely to interfere with patient's quality of life, since defibrillation function, which is the main mechanism by which ICDs can prolong a patient's life, has great potential to cause unfavorable psychological outcomes, compromising patient acceptance and adaptation to treatment [1], [2], [3], [4], [5], [6], [7].

Although there is no doubt about the role of the ICD in preventing sudden cardiac death, especially in patients with arrhythmogenic genetic disorders and ventricular dysfunction, international guidelines recommend that patients should be informed of all risks and benefits, including transient declines in quality of life after ICD shock therapies, preferably through a multidisciplinary approach, which should be maintained in the clinical follow-up phase, according to the evolution of each individual [8].

Patient device acceptance is a crucial factor in identifying the risk for unfavorable patient-reported outcomes. In this scenario, the Florida Patient Acceptance Survey (FPAS) was developed to measure patient acceptance of an implantable cardiac device for use both in clinical research and routine clinical practice [9]. In a short time, FPAS achieved great familiarity among international researchers, especially for the evaluation of ICD patients. Currently, this instrument has already been translated and validated in several countries such as Denmark [10], the Netherlands [11], Poland [12], China [13], Turkey [14], Colombia [15], and Japan [16], showing consistent results.

Originally, the FPAS instrument consists of 18 items that describe living with a cardiac device. Of all items, 15 are scored and contribute to the calculation of the questionnaire scores [9]. The validation of the FPAS instrument in the Netherlands, with a more representative sample, showed that an abbreviated version (FPAS-12 items) presented better indicators of psychometric adjustment compared to the complete version of the instrument [11].

Considering the importance of better understanding the level of patient acceptance of the cardiac device, the purpose of this study was to investigate the evidence of the validity of internal structure and reliability of the FPAS in a sample of Brazilian ICD patients, comparing the psychometric adjustment indicators of the complete (FPAS-18 item) and abbreviated (FPAS-12 item) versions.

### Study design

This is a psychometric study conducted at a tertiary cardiology academic hospital in Sao Paulo - Brazil.

### Florida patient acceptance survey (FPAS)

The FPAS instrument was developed in the United States in 2005 to assess the level of device acceptance of patients who had undergone an ICD or pacemaker implantation. According to the authors of FPAS, patient acceptance should be considered a device-specific component of the quality-of-life construct. Thus, the items of this questionnaire aim to assess the “psychological accommodation and understanding of the advantages and disadvantages of the cardiac device”, the recommendation of the device to others, and its benefits in terms of physical health, psychological, and social functioning [9].

The original instrument consists of 18 items, of which three are filler items and are not reflected in the calculation of the questionnaire scores (questions 9, 11, 16). The remaining 15 items are rated on a 5-point Likert scale ranging from 1 (strongly disagree) to 5 (strongly agree). The total score is determined by the sum of all items, transformed into a scale from 0 to 100 points, with a high score indicating better device acceptance [9]. The results of a psychometric investigation published in 2012 indicated that eliminating three items from the FPAS provided a better psychometric adjustment, which gave rise to an abbreviated version of the instrument, the FPAS 12-item [11].

### Cross-cultural adaptation of the FPAS instrument

The cross-cultural adaptation process of the FPAS followed international recommendations [17], [18], [19], [20]. The original instrument was translated into Brazilian Portuguese by two bilingual independent translators proficient in both the English and Portuguese languages. The versions prepared by the two independent translators were consolidated into a single version, after evaluation by a third independent translator. Working from the last version and totally blinded to the original version, two other bilingual translators performed the back-translations. The purpose of this step was to assess whether the version obtained reflected the same content as the original instrument.

Subsequently, all the material from the previous steps was then handed over to a multidisciplinary expert committee composed of physicians and nurses with extensive knowledge of cardiac pacing. The purpose of this expert committee was to analyze the adequacy of the translations and back-translations to determine whether the semantic, idiomatic, cultural, and conceptual equivalences had been maintained throughout the process.

After these steps, a prefinal version of the FPAS-Br was prepared by the research team and administered to a convenience sample of 20 ICD adult patients, of both genders, who agreed to participate in the pilot testing by signing the informed consent form. These patients were recruited during an outpatient clinic appointment and had the ICD implanted for at least 6 months. The pilot test was carried out to identify and correct possible translation issues. It was established that the translation would be revised or reformulated if less than 80% of the participants were able to understand the questionnaire items. Once all necessary changes and adjustments were completed, the final version of the FPAS-Br instrument was sent to the original author for a final check.

### Sample size

The determination of sample size for psychometric studies is habitually calculated based on the number of items of the instrument. A participant-item ratio of 10:1 is normally sufficient to allow adequate inference in confirmatory factor analysis [19,20]. Therefore, a minimum sample of 150 patients was established, allowing a ratio of 10:1 and 12:6 participants per item of the instrument, considering the 15 items that can be analyzed in the FPAS complete version and the 12 items in the abbreviated version, respectively.

### Participants

A convenience sample of 151 consecutive participants was enrolled in the study. Inclusion criteria were: (i) age 18 years or over; (ii) ICD implanted for at least 6 months; (iii) able to give informed consent. Patients with indications for cardiac transplantation and/or medical history of cognitive impairment or mental illness were not included in this study.

During outpatient care visits eligible patients were invited to participate in the study. After the consent was granted, the FPAS was self-completed by the participants. At the same time, demographics (age, race, sex, education level, marital status), clinical (underlying heart disease, comorbidities, functional class for heart failure, left ventricular ejection fraction, use of medications), and ICD information (indication, date of implantation, type of ICD, previous shock therapies) were collected directly on tablets, using electronic case report forms (eCRFs) developed in the Research Electronic Data Capture (REDCap) software [21] hosted at our Institution data center.

### Data analysis

We used exploratory factor analysis and confirmatory factor analysis for the evaluation of the validity of the internal structure of the FPAS-Br versions. Robust parallel analysis through the optimal implementation of parallel analysis with minimum rank factor analysis was performed for dimensionality testing [22,23]. The robustness of the test was determined from the association of bootstrap with sample extrapolation to 5000 cases. Factor extraction was performed with robust unweighted least squares, which reduces the residues of matrices [23,24].

The quality of the FPAS-Br versions was evaluated by the following psychometric parameters: explained variance of the model (60–70%), factor loading values (> 0.45), communalities (> 0.40), collinearity and multicollinearity problems (factor loads ranging from 0.80 to 0.85). The maintenance or withdrawal of an item was defined by the magnitude of the communalities, factor loads, sample size, the degree to which the item can measure the factor, and the lack of cross-loading [22], [23], [24], [25], [26].

The confirmatory factor analysis model adjustment indexes and their respective expected values were NNFI (Non-Normed Fit Index > 0.93), CFI (Comparative Fit Index > 0.94), GFI (Goodness Fit Index > 0.95), AGFI (Adjusted Goodness Fit Index > 0.93), RMSEA (Root Mean Square Error of Approximation ≤ 0.07) and RMSR (Root Mean Square of Residuals < 0.08) [24], [25], [26].

The reliability of the FPAS-Br was evaluated by Cronbach's alpha and McDonald's omega coefficients. The adoption of two indications aimed to increase the accuracy of the interpretation, since Cronbach's alpha coefficient may be affected by the nature of data distribution and by sample size [27].

The analyses were performed using SPSS 23 statistical package software and Factor 10.8.0.

### Final version of the FPAS-Br instrument

The cross-cultural adaptation process resulted in similar versions of the instrument, evidencing the good quality of the translations and the synthesis process carried out by the expert committee. Table 1 shows the instrument items in their English and Brazilian Portuguese versions.

**Table 1:** Original and Brazilian versions of the Florida Patient Acceptance Survey.

Item	Original version: FPAS	Brazilian version: FPAS-Br
	**Introduction**	**Introdução**
	We want to understand what it is like for you to live with a medical device. Below are some statements that describe living with a medical device. Please rate the extent to which you agree or disagree with each of the following statements by checking the most appropriate box.	Nós gostaríamos de saber como é para você viver com um aparelho implantado em seu corpo. Abaixo estão algumas afirmações que descrevem como é viver com um dispositivo cardíaco. Por favor, escolha o quanto você concorda ou discorda com cada uma das afirmações.
	**Questions**	**Perguntas**
1	Thinking about the device makes me depressed.	Fico deprimido só de pensar no meu aparelho.
2	When I think about the device, I avoid doing things I enjoy.	Quando eu penso no meu aparelho, eu evito fazer as coisas que eu gosto.
3	I avoid my usual activities because I feel disfigured by my device.	Eu evito minhas atividades rotineiras porque eu me sinto incomodado pelo meu aparelho.
4	It is hard for me to function without thinking about my device.	É difícil para mim viver sem pensar no meu aparelho.
5	My device was my best treatment option.	O aparelho foi a melhor opção de tratamento para a minha doença.
6	I am confident about my ability to return to work if I want to.	Eu tenho certeza de que tenho condições de voltar a trabalhar se eu quiser.
7	I am safer from harm because of my device.	Eu me sinto seguro em relação à minha doença por ter o meu aparelho implantado.
8	The positive benefits of this device out-weigh the negatives.	As vantagens de ter esse aparelho são maiores que as desvantagens.
9^a^	I have continued my normal sex life.	Eu continuei minha vida sexual normalmente.
10	I would receive this device again.	Eu colocaria este aparelho de novo.
11^a^	I know enough about my device.	Eu tenho conhecimento suficiente sobre o meu aparelho.
12^b^	I am careful when hugging or kissing my loved ones.	Eu tenho cuidado ao abraçar ou beijar as pessoas que eu gosto.
13	I have returned to a full life.	Eu voltei a ter uma vida totalmente normal.
14^b^	I feel that others see me as disfigured by my device.	Eu sinto que as outras pessoas me acham menos atraente por causa do meu aparelho.
15^b^	I feel less attractive because of my device.	Eu me sinto menos atraente por causa do meu aparelho.
16^a^	I am knowledgeable about how the device works and what it does for me.	Eu estou bem informado sobre a forma como o aparelho funciona e o que ele faz por mim.
17	I am not able to do things for my family the way I used to.	Eu não sou capaz de fazer as coisas para a minha família do mesmo jeito que fazia antes de ter o aparelho.
18	I am concerned about resuming my daily physical activities.	Eu tenho preocupações se posso voltar às minhas atividades físicas diárias.
	**Response options**	**Opções de resposta**
	Strongly disagree (1) Mostly disagree (2) Neither agree nor disagree (3) Mostly agree (4) Strongly agree (5)	Discordo totalmente (1) Discordo parcialmente (2) Nem concordo, nem discordo (3) Concordo parcialmente (4) Concordo totalmente (5)

a Items 9, 11, and 16 are filler items and do not reflect in the calculation of the instrument scores.

b Items 12, 14, and 15 were removed from the original version giving rise to the FPAS 12-item.^11^

### Sample characteristics

The sample (*n* = 151 ICD patients) had a mean age of 55.7 ± 14.1 years and was predominantly male. The baseline characteristics of the sample are described in Table 2.

**Table 2:** Demographic and clinical profile of the study participants (*n* = 151).

Characteristics	
Male sex, n (%)	97 (64.2)
Age (years), mean ± standard deviation	55.7 ± 14.1
White (race), n (%)	129 (85.4)
Education, n (%)	
Primary school (up to 9 years of schooling)	77 (51.0)
Secondary school (up to 12 years of schooling)	52 (34.4)
Higher education (over 12 years of schooling)	22 (14.6)
Marital status, n (%)	
Married	98 (64.9)
Single	22 (14.6)
Divorced	12 (7.9)
Widow	10 (6.6)
Stable union	9 (6.0)
Underlying heart disease, n (%)	
Chagas disease	46 (30.5)
Ischemic cardiomyopathy	38 (25.2)
Hypertrophic cardiomyopathy	22 (14.6)
Dilated cardiomyopathy	20 (13.2)
Brugada Syndrome	7 (4.6)
Congenital Long QT Syndrome	5 (3.3)
Right ventricular arrhythmogenic dysplasia	4 (2.6)
Idiopathic ventricular fibrillation	2 (1.3)
Sarcoidosis	2 (1.3)
Others	5 (3.3)
Heart failure functional class (New York Heart Association), n (%)	
I - II	128 (84.8)
III - IV	23 (15.2)
Left ventricular ejection fraction, mean ± standard deviation	41.2 ± 15.6
Comorbidities, n (%)	
None	44 (29.1)
Dyslipidemia	55 (36.4)
Hypertension	53 (35.1)
Coronary arterial disease	29 (19.2)
Atrial fibrillation	29 (19.2)
Diabetes	22 (14.6)
Chronic kidney disease	7 (4.6)
Chronic obstructive pulmonary disease	5 (3.3)
Use of medication, n (%)	
Betablockers	129 (85.4)
Antiarrhythmic drugs	89 (58.9)
Angiotensin-converting enzyme inhibitor	76 (50.3)
Diuretics	76 (50.3)
Antiplatelets	48 (31.8)
Oral anticoagulants	42 (27.8)
Angiotensin receptor blocker	33 (21.9)
Implantable cardioverter-defibrillator indication, n (%)	
Primary prophylaxis of sudden cardiac death	30 (19.9)
Secondary prophylaxis of sudden cardiac death	121 (80.1)
Implantable cardioverter-defibrillator type, n (%)	
Atrioventricular	70 (46.4)
Ventricular	62 (41.1)
Cardiac resynchronization	19 (12.6)
Time of device implantation (years), mean ± standard deviation	6.7 ± 4.4
Implantable cardioverter-defibrillator therapies, n (%)	
Received shock therapies	91 (60.3)
Never received shock therapies	60 (39.7)

## Method validation

### Internal structure validity and reliability of the FPAS-Br

Factorial loads of the FPAS-Br 18-item ranged from 0.19 to 0.90, presenting two items that did not reach the minimum recommended value. The communalities ranged from 0.10 to 0.83, and six items had values below 0.40. These results directly affected the explained variance of the model (58.2%), which did not reach the recommendation of 60%.

In the analysis of the FPAS-Br 12-item, the factorial loads ranged from 0.37 to 0.90, and the communalities ranged from 0.22 to 0.84. In both indicators, two items did not reach the minimum recommended values. Despite this, the explained variance of this model was adequate (65.2%). These data show that the FPAS-Br 12-item presented better indicators, while the FPAS-Br 18-item had an average factorial load of 0.66 and communalities of 0.49; in the FPAS-Br 12-item, these averages were 0.73 and 0.58, respectively. (Table 3)

**Table 3:** Exploratory factor analysis of the FPAS-Br versions: factorial loads and communalities (*n* = 151).

Item	FPAS-Br 18-item			FPAS-Br 12-item		
	Factorial loads		Communalities	Factorial loads		Communalities
	Factor 1	Factor 2		Factor 1	Factor 2	
1	0.86		0.74	0.78		0.61
2	0.79		0.62	0.83		0.69
3	0.77		0.62	0.82		0.65
4	0.84		0.71	0.87		0.76
5		0.81	0.65		0.88	0.76
6		0.19	0.10		0.38	0.22
7		0.88	0.79		0.89	0.82
8		0.90	0.83		0.90	0.84
10		0.70	0.49		0.73	0.53
12	0.55		0.30			
13		0.32	0.16		0.37	0.23
14	0.69		0.48			
15	0.57		0.32			
17		0.52	0.27		0.64	0.41
18		0.54	0.29		0.67	0.45

Items 9, 11, and 16 are filler items and do not reflect in the calculation of the instrument scores. Therefore, these items were not included in the psychometric analyses. Items 12, 14, and 15 are not included in the FPAS 12-item.

Factor 1= negative aspects of cardiac device acceptance; Factor 2= positive aspects of cardiac device acceptance.

The Kaiser-Meyer-Olkin index, Bartlett's test of sphericity, and the matrix determinant revealed a significant correlation between the items for both versions of the FPAS-Br instrument, indicating that the data fulfilled the assumptions for carrying out a factor analysis. (Table 4)

**Table 4:** Comparison of the models of the FPAS-Br 18-item and FPAS-Br 12-item.

Psychometric analysis	FPAS-Br 18-item	FPAS-Br 12-item	Recommendations [22], [23], [24], [25], [26]
**Exploratory Factorial Analysis**			
Matrix determinant	0.004	0.001	*P* < 0.005
Bartlett	779.2 (df=105) *P*< 0.001	588.9 (df= 66) *P* < 0.001	*P* < 0.005
KMO (Kaiser-Meyer-Olkin)	0.78	0.78	> 0.60
Dimensions (Parallel Analysis)	2	2	
Explained variance by Eigenvalues	54.6%	64.5%	> 60%
Explained variance (Parallel Analysis)	58.2%	65.2%	> 60%
**Dimensionality**			
Unidimensional Congruence (UNICO)	0.74	0.76	Unidimensional > 0.95
Explained Common Variance (ECV)	0.62	0.61	Unidimensional > 0.85
Mean of item residual absolute loading (MIREAL)	0.32	0.42	Unidimensional < 0.30
**Confirmatory Factorial Analysis**			
Non-Normed Fit Index (NNFI)	0.95	0.97	> 0.93
Comparative Fit Index (CFI)	0.96	0.97	> 0.94
Goodness of Fit Index (GFI)	0.94	0.96	> 0.95
Adjusted Goodness of Fit Index (AGFI)	0.92	0.96	> 0.93
Root Mean Square of Residuals (RMSR)	0.10	0.07	≤ 0.07
Standardized Cronbach's Alpha	0.84	0.85	Excellent ≥ 0.85
McDonald's Omega	0.76	0.81	Excellent ≥ 0.85
Construct reliability G-H Latent index (> 0.80)	Factor 1 = 0.92; 0.68 Factor 2 = 0.92; 0.49	Factor 1 = 0.93; 0.52 Factor 2 = 0.92; 0.76	Excellent ≥ 0.80

The parallel analysis, used to test the dimensionality of the instrument, indicated the presence of a two-dimensional model in both instrument versions, reflecting negative (Factor 1) and positive (Factor 2) aspects. This two-dimensional model was confirmed by complementary indexes of the dimensionality assessment as shown in Table 4. The confirmatory factor analysis showed a good fit to the two-dimensional model, with values comparable to those recommended in the literature for practically all the quality indicators, except for the RMSR (Root Mean Square of Residuals) in the FPAS-Br 18-item, which was above the recommended values.

We found satisfactory evidence of the reliability of the FPAS-Br instrument, with Cronbach's alpha coefficients of 0.84 and 0.85 and McDonald's Omega coefficients of 0.76 and 0.81 for the FPAS-Br 18-item and FPAS-Br 12-item versions, respectively. (Table 4)

### Study implications

The adoption of patient-reported outcomes as a tool to measure treatment results from the patient's perspective has become increasingly frequent. In the current era of patient-centered care, the incorporation of this approach into routine practice has been considered an important metric of better clinical practices [28,29]. Among the outcomes reported by patients, metrics of quality of life, symptoms, functionality, anxiety, patient acceptance, psychosocial adaptation, satisfaction, well-being, and preferences stand out.

In the present study, we described the cross-adaptation process and validation of an instrument designed to measure the level of patient acceptance of the cardiac device, following international methodological standards [17], [18], [19]. The final version of the FPAS in Brazilian Portuguese (FPAS-Br) presented conceptual, semantic, cultural, and measurement equivalences compared to the original items in English [9].

According to the authors of the FPAS instrument, the level of device acceptance is a crucial aspect to identify patients at high risk of unfavorable psychological outcomes, and there is an inverse relationship between device acceptance and the clinical presentation of depression and anxiety [9], [10], [11]. In the international scenario, the FPAS has been widely used in different contexts, as it has good sensitivity to identify the level of acceptance of the cardiac implantable electronic devices with a reduced time to fill it out the questionnaire [[10], [11], [12], [13], [14], [15], [16],^30^]. Although there is no validated cut-off to categorize device acceptance into poor and good, previous studies have used the lower tercile of the total score to categorize patients into “no acceptors” and “acceptors” [9,10,30].

In the original FPAS publication, the authors presented an instrument with 15 scored items comprising four factors (“Return to Function”, “Device-Related Distress”, “Positive Appraisal”, and “Body Image Concerns”). The instrument also presents three filler items (items numbered: 9, 11, 16), thus yielding 18 items in the final FPAS [9]. Initial psychometric analyses using factor scores showed that a higher-order factor analysis procedure resulted in a single factor supporting the use of a single score, reflecting the total FPAS score. Subsequently, a shortened 12-item, three-factor version of the FPAS (“Return to Function”, “Device-Related Distress”, “Positive Appraisal”) was shown to be a valid and internally consistent instrument in a Dutch ICD cohort, accounting for 63.6% of the explained variance of the model [11].

Considering the existence of the 18-item and 12-item versions of the FPAS, in the present study, we compared the psychometric adjustment indicators of these two models: the complete and the abbreviated versions. The analysis of the different indicators allowed us to state that the 12-item version proved to be superior to the 18-item version, presenting better results in the factorial load (average= 0.73 versus 0.66), in the communalities (average= 0.58 versus 0.49), in the explained variance of the model (65.2% versus 58.2%), and in different adjustment parameters of the factor analysis as described in Table 4. The analyses used to test the dimensionality of the FPAS, however, pointed to a bi-dimensional instrument in both versions, allowing a grouping of items into negative (Factor 1) and positive (Factor 2) aspects of the life of a patient with a cardiac device, differing from the original model proposed, which has four dimensions (two related to positive aspects and two related to negative aspects). Similar to the original model, our results also supported the use of a total single score, which has been the most commonly used method in different publications [[10], [11], [12], [13], [14], [15], [16],34]. Regarding the reliability of the FPAS-Br, the analyses revealed the precision of both versions, which was confirmed by adequate Cronbach's alpha and McDonald's Omega coefficients.

The limitations of the present study must be acknowledged. Although the population studied is larger than the samples of several previous publications that have used the FPAS, further studies with more robust samples are crucial for the consolidation of its validity and for attesting its stability in different scenarios, as well as in patients with other types of cardiac devices, such as pacemakers and cardiac resynchronization therapy. Future studies evaluating the association of the FPAS-Br scores with the occurrence of ICD shock therapies and other clinical parameters will be useful to identify factors that can predict device acceptance and thus allow the establishment of personalized interventions for these patients.

In conclusion, the two versions of the FPAS-Br instrument showed consistent evidence of internal structure validity and reliability. Of the two, the FPAS-Br 12-item version seems to be more suitable for use in clinical practice and research due to its brevity, in addition to its better psychometric adjustment.

The main contribution of the present study refers to the availability of a valid and reliable instrument for the assessment of acceptance and psychosocial adaptation of Brazilian patients with ICD, which can be used either in routine care or in research scenarios.

## Ethics statements

The study was approved by the institutional Ethics Committee and complied with the Declaration of Helsinki and good clinical practice guidelines. All participants provided written informed consent before their inclusion in the study.

## CRediT author statement

**Katia Regina Silva:** Conceptualization, Methodology, Software, Funding acquisition, Writing-Original draft preparation, Writing- Reviewing and Editing, **Roberto Costa:** Methodology, Writing- Reviewing and Editing, **Flávio Rebustini:** Methodology, Software, Formal analysis, Writing-Original draft preparation, **Giovanna Regina Garcia de Oliveira Melo:** Data collection, **Laísa de Arruda Silva:** Data curation, **Sarah Caroline Martins Saucedo:** Data curation, **Samuel Sears:** Methodology, Writing- Reviewing and Editing.

## Declaration of Competing Interest

The authors declare that they have no known competing financial interests or personal relationships that could have appeared to influence the work reported in this paper.

## Data Availability

Data will be made available on request.

## Appendix Supplementary materials

Image, application 1
