# Supplementary material for: Validity and reliability of the Brazilian Portuguese version of the Florida Patient Acceptance Survey for patients with implantable cardioverter defibrillators
Source: MethodsX. 2023 Jun 27;11:102272. doi: 10.1016/j.mex.2023.102272 (PMC10719526; doi:10.1016/j.mex.2023.102272)
Supplement: Supplementary file 1 [file mmc1.pdf]

Versão Brasileira do Instrumento Florida Patient Acceptance Survey (FPAS-Br)

Nós gostaríamos de saber como é para você viver com um aparelho implantado em seu corpo. Abaixo estão algumas afirmações que descrevem como é viver com um dispositivo cardíaco. Por favor, escolha o quanto você CONCORDA ou DISCORDA com cada uma das afirmações.

FPAS-Br versão completa (18 itens)		FPAS-Br versão abreviada (12 itens)	
1.	Fico deprimido só de pensar no meu aparelho.	1.	Fico deprimido só de pensar no meu aparelho.
2.	Quando eu penso no meu aparelho, eu evito fazer as coisas que eu gosto.	2.	Quando eu penso no meu aparelho, eu evito fazer as coisas que eu gosto.
3.	Eu evito minhas atividades rotineiras porque eu me sinto incomodado pelo meu aparelho.	3.	Eu evito minhas atividades rotineiras porque eu me sinto incomodado pelo meu aparelho.
4.	É difícil para mim viver sem pensar no meu aparelho.	4.	É difícil para mim viver sem pensar no meu aparelho.
5.	O aparelho foi a melhor opção de tratamento para a minha doença.	5.	O aparelho foi a melhor opção de tratamento para a minha doença.
6.	Eu tenho certeza de que tenho condições de voltar a trabalhar se eu quiser.	6.	Eu tenho certeza de que tenho condições de voltar a trabalhar se eu quiser.
7.	Eu me sinto seguro em relação à minha doença por ter o meu aparelho implantado.	7.	Eu me sinto seguro em relação à minha doença por ter o meu aparelho implantado.
8.	As vantagens de ter esse aparelho são maiores que as desvantagens.	8.	As vantagens de ter esse aparelho são maiores que as desvantagens.
9.	*Eu continuei minha vida sexual normalmente.		
10.	Eu colocaria este aparelho de novo.	9.	Eu colocaria este aparelho de novo.
11.	*Eu tenho conhecimento suficiente sobre o meu aparelho.		
12.	Eu tenho cuidado ao abraçar ou beijar as pessoas que eu gosto.		
13.	Eu voltei a ter uma vida totalmente normal.	10.	Eu voltei a ter uma vida totalmente normal.
14.	Eu sinto que as outras pessoas me acham menos atraente por causa do meu aparelho.		
15.	Eu me sinto menos atraente por causa do meu aparelho.		

FPAS-Br versão completa (18 itens)	FPAS-Br versão abreviada (12 itens)
16. *Eu estou bem informado sobre a forma como o aparelho funciona e o que ele faz por mim.	
17. Eu não sou capaz de fazer as coisas para a minha família do mesmo jeito que fazia antes de ter o aparelho.	11. Eu não sou capaz de fazer as coisas para a minha família do mesmo jeito que fazia antes de ter o aparelho.
18. Eu tenho preocupações se posso voltar às minhas atividades físicas diárias.	12. Eu tenho preocupações se posso voltar às minhas atividades físicas diárias.
**Opções de resposta**<br>1 - Discordo totalmente<br>2 - Discordo parcialmente<br>3 - Nem concordo, nem discordo<br>4 - Concordo parcialmente<br>5 - Concordo totalmente	

* Os itens 9, 11, 16 do FPAS-18 são itens de preenchimento opcional e não refletem no cálculo dos escores do instrumento.

Cálculo dos Escores do Instrumento Florida Patient Acceptance Survey (FPAS)

- O escore total do FPAS é obtido pela soma de todos os itens, transformada numa escala de 0 a 100 pontos.
- A pontuação total do FPAS é positivamente correlacionada com a aceitação do dispositivo, ou seja, pontuações mais baixas refletem menor aceitação ao dispositivo cardíaco.
- A versão brasileira do instrumento apresenta dois domínios: aspectos negativos e aspectos positivos relacionados à aceitação do dispositivo cardíaco.

FPAS-Br versão completa (18 itens)	FPAS-Br versão abreviada (12 itens)
Os itens 1, 2, 3, 4, 12, 14, 15, 17, 18 são pontuados de forma reversa e usados em todas as análises como pontuação reversa.	Os itens 1, 2, 3, 4, 11, 12 são pontuados de forma reversa e usados em todas as análises como pontuação reversa.
**Escore Total Bruto (raw)**<br>1® + 2® + 3® + 4® + 5 + 6 + 7 + 8 + 10 + 12® + 13 + 14® + 15® + 17® + 18®<br><br>**Escore Final (0 a 100 pontos)**<br>(raw – 15)/60*100	**Escore Total Bruto (raw)**<br>1® + 2® + 3® + 4® + 5 + 6 + 7 + 8 + 9 +10 + 11® + 12®<br><br>**Escore Final (0 a 100 pontos)**<br>(raw – 12)/ 48*100
Domínio 1 - Aspectos negativos relacionados à aceitação do dispositivo cardíaco<br>Itens: 1, 2, 3, 4, 12, 14, 15<br>**Escore Bruto – Domínio 1 (raw)**<br>1® + 2® + 3® + 4® + 12® + 14® + 15®<br>**Escore Final – Domínio 1 (0 a 100 pontos)**<br>100- (raw – 7)/28*100	***Domínio 1 - Aspectos negativos relacionados à aceitação do dispositivo cardíaco***<br>Itens: 1, 2, 3, 4<br>**Escore Bruto – Domínio 1 (raw)**<br>1® + 2® + 3® + 4®<br>**Escore Final – Domínio 1 (0 a 100 pontos)**<br>100 - (raw – 4)/16*100
Domínio 2 - Aspectos positivos relacionados à aceitação do dispositivo cardíaco<br>Itens: 5, 6, 7, 8, 10, 13, 17, 18<br>**Escore Bruto – Domínio 2 (raw)**<br>5 + 6 + 7 + 8 + 10 + 13 + 17 +18<br>**Escore Final – Domínio 2 (0 a 100 pontos)**<br>(raw – 8)/32*100	***Domínio 2 - Aspectos positivos relacionados à aceitação do dispositivo cardíaco***<br>Itens: 5, 6, 7, 8, 9, 10, 11, 12<br>**Escore Bruto – Domínio 2 (raw)**<br>5 + 6 + 7 + 8 + 9 + 10 + 11 +12<br>**Escore Final – Domínio 2 (0 a 100 pontos)**<br>(raw – 8)/32*100

Versão Brasileira do Instrumento Florida Patient Acceptance Survey (FPAS-Br)
Versão Abreviada (12-itens)

Nós gostaríamos de saber como é para você viver com um aparelho implantado em seu corpo. Abaixo estão algumas afirmações que descrevem como é viver com um dispositivo cardíaco. Por favor, escolha o quanto você CONCORDA ou DISCORDA com cada uma das afirmações.

1. Fico deprimido só de pensar no aparelho.
   1 - Discordo totalmente
   2 - Discordo parcialmente
   3 - Nem concordo, nem discordo
   4 - Concordo parcialmente
   5 - Concordo totalmente

2. Quando eu penso no meu aparelho, eu evito fazer as coisas que eu gosto.
   1 - Discordo totalmente
   2 - Discordo parcialmente
   3 - Nem concordo, nem discordo
   4 - Concordo parcialmente
   5 - Concordo totalmente

3. Eu evito minhas atividades rotineiras porque eu me sinto incomodado pelo meu aparelho.
   1 - Discordo totalmente
   2 - Discordo parcialmente
   3 - Nem concordo, nem discordo
   4 - Concordo parcialmente
   5 - Concordo totalmente

4. É difícil para mim viver sem pensar no meu aparelho.
   1 - Discordo totalmente
   2 - Discordo parcialmente
   3 - Nem concordo, nem discordo
   4 - Concordo parcialmente
   5 - Concordo totalmente

5. O aparelho foi a melhor opção de tratamento para a minha doença.
   1 - Discordo totalmente
   2 - Discordo parcialmente
   3 - Nem concordo, nem discordo
   4 - Concordo parcialmente
   5 - Concordo totalmente

6. Eu tenho certeza de que tenho condições de voltar a trabalhar se eu quiser.
   1 - Discordo totalmente
   2 - Discordo parcialmente
   3 - Nem concordo, nem discordo
   4 - Concordo parcialmente

5 - Concordo totalmente

7. Eu me sinto seguro em relação à minha doença por ter meu aparelho implantado.
   1 - Discordo totalmente
   2 - Discordo parcialmente
   3 - Nem concordo, nem discordo
   4 - Concordo parcialmente
   5 - Concordo totalmente

8. As vantagens de ter este aparelho são maiores que as desvantagens.
   1 - Discordo totalmente
   2 - Discordo parcialmente
   3 - Nem concordo, nem discordo
   4 - Concordo parcialmente
   5 - Concordo totalmente

9. Eu colocaria este aparelho de novo.
   1 - Discordo totalmente
   2 - Discordo parcialmente
   3 - Nem concordo, nem discordo
   4 - Concordo parcialmente
   5 - Concordo totalmente

10. Eu voltei a ter uma vida totalmente normal.
   1 - Discordo totalmente
   2 - Discordo parcialmente
   3 - Nem concordo, nem discordo
   4 - Concordo parcialmente
   5 - Concordo totalmente

11. Eu não sou capaz de fazer as coisas para minha família do mesmo jeito que costumava fazer antes de ter o aparelho.
   1 - Discordo totalmente
   2 - Discordo parcialmente
   3 - Nem concordo, nem discordo
   4 - Concordo parcialmente
   5 - Concordo totalmente

12. Eu tenho preocupações se posso voltar às minhas atividades físicas diárias.
   1 - Discordo totalmente
   2 - Discordo parcialmente
   3 - Nem concordo, nem discordo
   4 - Concordo parcialmente
   5 - Concordo totalmente
